# Deletion of *Seipin* Attenuates Vascular Function and the Anticontractile Effect of Perivascular Adipose Tissue

**DOI:** 10.3389/fcvm.2021.706924

**Published:** 2021-08-02

**Authors:** Mengyu Wang, Junhui Xing, Mengduan Liu, Mingming Gao, Yangyang Liu, Xiaowei Li, Liang Hu, Xiaoyan Zhao, Jiawei Liao, George Liu, Jianzeng Dong

**Affiliations:** ^1^Department of Cardiology, Henan Key Laboratory of Hereditary Cardiovascular Diseases, The First Affiliated Hospital of Zhengzhou University, Zhengzhou, China; ^2^Laboratory of Lipid Metabolism, Hebei Medical University, Shijiazhuang, China; ^3^Department of Cardiology, Institute of Cardiovascular Diseases, First Affiliated Hospital of Dalian Medical University, Dalian, China; ^4^Key Laboratory of Molecular Cardiovascular Sciences, Peking University Health Science Center, School of Basic Medical Sciences, Institute of Cardiovascular Sciences, Ministry of Education, Beijing, China; ^5^Department of Cardiology, National Clinical Research Centre for Cardiovascular Diseases, Beijing Anzhen Hospital, Capital Medical University, Beijing, China

**Keywords:** *Seipin*, lipodystrophy, PVAT, vascular function, anticontractile function

## Abstract

*Seipin* locates in endoplasmic reticulum (ER) and regulates adipogenesis and lipid droplet formation. Deletion of *Seipin* has been well-demonstrated to cause severe general lipodystrophy, however, its role in maintaining perivascular adipose tissue (PVAT) and vascular homeostasis has not been directly assessed. In the present study, we investigated the role of *Seipin* in mediating the anticontractile effect of PVAT and vascular function. *Seipin* expression in PVAT and associated vessels were detected by qPCR and western-blot. *Seipin* is highly expressed in PVAT, but hardly in vessels. Structural and functional alterations of PVAT and associated vessels were compared between *Seipin*^−/−^ mice and WT mice. In *Seipin*^−/−^ mice, aortic and mesenteric PVAT were significantly reduced in mass and adipose-derived relaxing factors (ADRFs) secretion, but increased in macrophage infiltration and ER stress, as compared with those in WT mice. Aortic and mesenteric artery rings from WT and *Seipin*^−/−^ mice were mounted on a wire myograph. Vasoconstriction and vasodilation were studied in vessels with and without PVAT. WT PVAT augmented relaxation but not *Seipin*^−/−^ PVAT, which suggest impaired anticontractile function in PVAT of *Seipin*^−/−^ mice. Thoracic aorta and mesenteric artery from *Seipin*^−/−^ mice had impaired contractility in response to phenylephrine (PHE) and relaxation to acetylcholine (Ach). In conclusion, *Seipin* deficiency caused abnormalities in PVAT morphology and vascular functions. Our data demonstrated for the first time that *Seipin* plays a critical role in maintaining PVAT function and vascular homeostasis.

## Introduction

*Seipin*, an endoplasmic reticulum (ER) membrane protein regulating adipogenesis and lipid droplet formation, is the culprit gene for human Berardinelli-Seip congenital lipodystrophy type 2 (BSCL2) (1, 2). BSCL2 is an autosomal recessive disorder, which is characterized by the severe loss of adipose tissue, hypertriglyceridemia, fatty liver and insulin resistance (3). Recent studies have demonstrated that *Seipin* regulates adipocyte lipolysis in addition to differentiation (2, 4). Besides, *Seipin* functions in the metabolism of phospholipids and therefore determines the size and distribution of lipid droplets (5–7). Most researches focused on metabolic disorders of *Seipin* deficiency. And, a small number of studies was found to focus on cardiovascular diseases such as atherogenesis, myocardial hypertrophy, heart remodeling and hypertension (8–10). However, whether *Seipin* regulates vascular activity remains obscure.

PVAT is tightly adherent to almost all blood vessels, including the aorta and arteries such as carotid, coronary, and mesenteric arteries, and it has long been considered as providing mechanical support for vessels. Recent studies have suggested that crosstalk between PVAT and blood vessels is vital for vascular homeostasis (11, 12). Now, it is increasingly accepted that PVAT also secrets a large number of biologically active molecules (13–15). PVAT can release not only adipocyte-derived relaxing factors (ADRFs) (e.g., leptin and adiponectin) but also adipocyte-derived contracting factors (ADCFs) (e.g., Ang ll and superoxide anions) and inflammatory factors (e.g., MCP-1 and IL-6). The function and structure of vascular wall, including chronic inflammation and vascular reactivity regulation, might be influenced by these molecules. Accumulating data indicates that PVAT has anticontractile effect on arteries and regulates vascular reactivity. ADRFs is likely not a singular entity. ADRFs such as NO, Ang (1–7), hydrogen sulfide, leptin and adiponectin may mediate the PVAT vasorelaxant effect in part (16–18). However, in several pathophysiological conditions, PVAT function may be altered. It has also been demonstrated that the anticontractile effect is reduced under the obese condition (19, 20). In obese condition, PVAT depot greatly expands accompanied with macrophages accumulation, which contributes to an inflammatory phenotype switch and may be implicated in vascular dysfunction (21, 22). Lipodystrophy, the opposite of obesity, is accompanied with many metabolic disorders in human patients, such as severe hypertriglyceridemia, hepatic steatosis, insulin resistance and atherosclerosis (23–25). However, little is known about the PVAT function and vascular activity regulation in state of lipodystrophy.

In the current study, we demonstrated impaired PVAT and vascular function in lipodystrophic *Seipin*^−/−^ mice. Our data supports the idea that *Seipin* is required for maintaining normal PVAT morphology and vascular homeostasis, and provides a direct evidence of the tight correlation between PVAT dysfunction and vascular activity.

## Materials and Methods

### Animals

Lipodystrophic *Seipin*^−/−^ mice on C57 background were generated as described previously (26) and WT littermates were used as controls. All mice were maintained on a 12-h light/12-h dark cycle with free access to water and food. Only males were included in the experiments. All experiments involving mice were accorded with the Institutional Animal Care Research Advisory Committee of the National Institute of Biological Science (NIBS) and approved by the Animal Care Committee of Zhengzhou University. Mice at 6 months old were anesthetized with pentobarbital sodium (40 mg/kg, i.p) and arterial blood pressure were measured by tail-cuff method.

### Blood Analysis

Blood was obtained by retro-orbital bleed. Plasma total cholesterol (TC), triglyceride (TG), glucose were detected using enzymatic methods (Sigma-Aldrich kits). Plasma insulin, leptin and adiponectin, tumor necrosis factor-α (TNF-α) and interleukin-6 (IL-6) were measured by ELISA (GPO-Trinder kit, Sigma-Aldrich). Free fatty acids (FFA) were measured by a colorimetric assay (Wako Chemical, Osaka, Japan).

### Glucose and Insulin Tolerance Tests

Mice were fasted for 16 or 4 h, respectively, followed by intraperitoneal injection of glucose (2 g/kg body weight; Abbott) or insulin (0.75 mIU/g body weight; Humulin). Blood samples were collected before (time 0) and at 15, 30, 60, 90, and 120 min after injection for glucose measurement (6).

### Histological Analysis

Mice were sacrificed at 6 months old. PVAT surrounding thoracic aorta and mesenteric arteries were removed and weighed, respectively. PVAT and aorta were then fixed in 10% buffered formalin and embedded in paraffin. Sections (2 um) were stained with hematoxylin and eosin, and also stained with Gomrori's aldehyde-fuchsin staining and Sirius red for microscopic observation of elastic fiber and collagen fiber changes. Macrophage infiltration in PVAT were visualized by immunochemical staining with macrophage antigen-2 (Mac-2) antibody (Santa Cruz Biotechnology, Dallas, TX).

### Electron Microscopy

For electron microscopy, blood vessels were fixed in 2.5% glutaraldehyde, and post fixed in 1% osmium tetroxide. Tissue slices were dehydrated with the different concentration ethanol and acetone, and embedded in Epon 812 resin. Ultrathin sections were stained with uranyl acetate and lead citrate and visualized witha JEOL 1230 transmission electron microscope (JEOL, Tokyo, Japan).

### Gene Expression Analysis

Total RNA of PVAT and aorta from 6-months-old *Seipin*^−/−^ and WT mice was extracted using Trizol reagent (Invitrogen, Carlsbad, CA). First-strand cDNA was generated by using a RT Kit (Invitrogen). Quantitative RT-PCR was performed using the Mx3000 Multiplex Quantitative PCR System (Stratagene). mRNA quantity was determined applying PCR protocols based on Eva Green detection and using primer sets shown in Table 1. All samples were quantitated by the comparative C_T_ method normalizing with GAPDH from the same sample of RNA.

**Table 1:** Primer list for quantitative real-time PCR.

**Gene name**	**Forward primer (5′-3′)**	**Reverse primer (5′-3′)**
*Seipin*	TCAATGACCCACCAGTC	AAGGAGCCATAGAGGACC
F4/80	TTTCCTCGCCTGCTTCTTC	CCCCGTCTGTATTCAACC
Mac2	CCTATGACCTGCCCTTGC	CCCAGTTGGCTGATTTCC
TNFα	CTGTGAAGGGAATGAATGTT	CAGGGAAGAATCTGGAAAGGTC
MCP1	TCCCAATGAGTAGGCTGGA	AAGTGCTTGAGGTGGTTGT
IL-1β	AGGCTCCGAGATGAACAA	AAGGCATTAGAAACAGTCC
Mgl1	TGAGAAAGGCTTTAAG AACTGGG	GACCACCTGTAGTGATGTGGG
Mgl2	GGATGGGACCGACTTTGA	GTGGGCTGAGCTGGCTTT
TGF-β1	GGCGGTGCTCGCTTTGTA	TCCCGAATGTCTGACGTAT
Arg1	AAGACAGCAGAGGAGGTG	AGTCAGTCCCTGGCTTAT
Ym1	GTAATGAGTGGGTTGGTT	AGTAGATGTCAGAGGGAAA
Col1a1	CGCCATCAAGGTCTACTGC	GAATCCATCGGTCATGCTCT
Col6a1	CACTCAACGGGACACGAC	AGATACCTGGCCGACCTT
Col3a1	GGCAGTGATGGGCAACCT	TCCCTTCGCACCGTTCTT
Elastin	GCAGCCCCTAACCAGAAACT	CCCACAAAGAAGAAGCAC
CHOP	TCCCTGCCTTTCACCTTG	CGTTCTCCTGCTCCTTCTC
GRP78	ACTTGGGGACCACCTATTCCT	ATCGCCAATCAGACGCTCC
GRP94	TCGTCAGAGCTGATG ATGAAGT	GCGTTTAACCCATCCAACTGAAT
mXBP1	AGCAGCAAGTGGTGGATTTG	GAGTTTTCTCCCGTAAAAGCTGA
ICAM1	GTGATGCTCAGGTATCCATCCA	CACAGTTCTCAAAGCACAGCG
VCAM1	AGTTGGGGATTCGGTTGTTCT	CCCCTCATTCCTTACCACCC
IL-6	TAGTCCTTCCTACCCC AATTTCC	TTGGTCCTTAGCCACTCCTTC

### Western Blot Analysis

PVAT and aortas tissues were homogenized in RIPA buffer, and the protein content was determined using a bicinchoninic acid protein assay kit (Pierce, Rockford, IL) as previously described (8). Immunoblotting was performed using the antibodies against *Seipin* (Abnova, Taipei, Taiwan), Mac2 (Santa Cruz, CA, USA), BIP/GRP78, PDI, PERK, phospho-PERK (Thr980), eIF2a and phospho-eIF2a (Ser51) (Cell Signaling, Danver, MA, USA), and GAPDH (Millipore, Billerica, MA). The examined proteins were detected using an Odyssey V3.0 image scanning (Li-COR, Inc., Lincoln, NE, USA). The protein bands were analyzed using densitometry, and arbitrary densitometry units were quantified are expressed as mean ± SEM.

### Vasoactivity Analysis

Experiments were performed as described before (27). Mice at 6 months old were killed by cervical dislocation. The thoracic aorta and second-order branches of mesenteric arteries were isolated, placed in cold (4°C) Krebs-Ring bicarbonate solution (in mmol/L: NaCl 118.6; KCl 4.7; CaCl_2_ 2.5; MgSO_4_ 1.2; KH_2_PO_4_ 1.2; NaHCO_3_ 25.1; EATANA_2_Ca 0.026; glucose 10.1). Arterial rings (2 mm long) with or without PVAT were dissected, and care was taken not to damage the endothelium during preparation. Arterial rings were mounted in a myograph system and contracted with KCL. The rings were exposed to phenylephrine (PE, 10^−9^-10^−5^ mol/L, Sigma-Aldrich), Constriction response to phenylephrine was assessed in WT and *Seipin*^−/−^ mice with PVAT or without PVAT, and the results were expressed as contraction percentage of maximum contraction to KCL. Relaxation responses to acetylcholine (Ach, 10^−9^-10^−5^ mol/L, Sigma-Aldrich) and sodium nitroprusside (SNP, 10^−9^-10^−5^ mol/L, Sigma-Aldrich) were performed after the aortic rings were pre-contracted with phenylephrine, and the results were expressed as percentage of the maximum response to acetylcholine and sodium nitroprusside. Aortas with PVAT acted as donor vessels and pre-contracted by phenylephrine. And aortas without PVAT acted as receptor vessels and also treated with equal concentration of phenylephrine. Five milliliter volume of culture medium of different donors were changed into receptor aorta, and the constriction response was recorded.

### Statistics

All data are presented as means ± SEM. Vascular function experiments were analyzed with ANOVA for repeated measurements followed by a Bonferroni *post-hoc* test. Comparisons between groups for remaining experiments were analyzed by Student's *t*-test. A value of *P* < 0.05 was considered statistically significant. All data were analyzed with Graphpad Prism 6.0.

## Results

### Insulin Resistance in *Seipin*^–/–^ Mice

**Figure 1:**
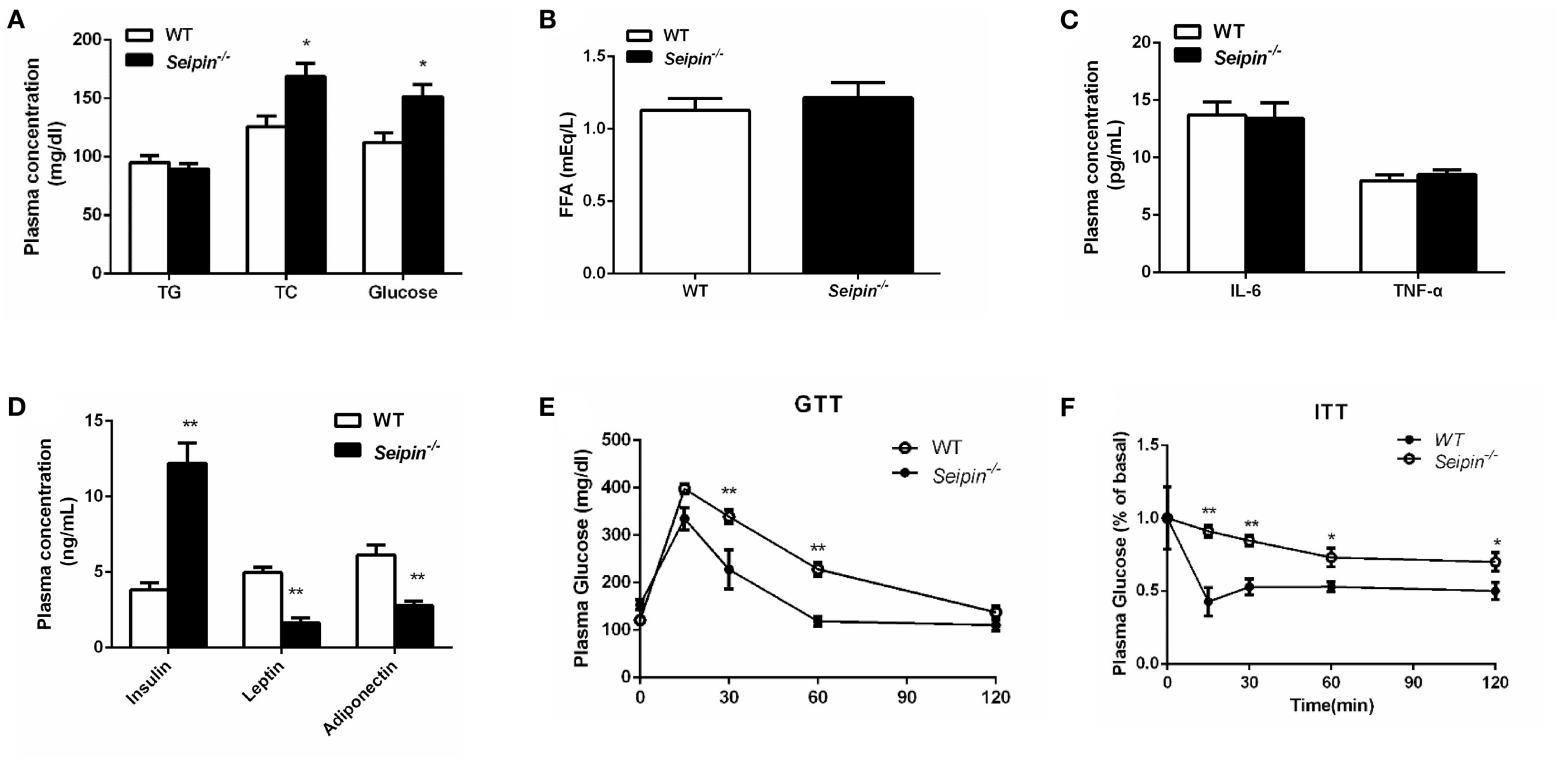
Insulin resistance in WT and *Seipin* deleted mice. **(A)** Plasma triglyceride (TG), total cholesterol (TC), glucose content in mice fasted for 4 h. **(B)** Free fatty acid (FFA) in mice fasted for 4 h. **(C)** Plasma levels of IL-6, TNF-α in mice fasted for 4 h. **(D)** Plasma insulin, leptin, adiponectin content in mice fasted for 4 h. **(E)** Glucose tolerance tests and insulin tolerance tests **(F)** performed on 6-month-old *Seipin*^−/−^ and WT mice. Data are expressed as mean ± SEM. *N* = 6. ^*^P < 0.05, ^**^P < 0.01 for *Seipin*^−/−^ mice vs. WT.

Total cholesterol (TC), glucose and insulin levels were significantly elevated as compared with WT mice in the fed state (Figures 1A,D). Plasma leptin and adiponectin levels were markedly decreased in *Seipin*^−/−^ mice (Figure 1D). Plasma levels of triglycerides (TG), free fatty acid (FFA), tumor necrosis factor-α (TNF-α) and interleukin-6 (IL-6) were not significantly altered (Figures 1A–C). We evaluated glucose homeostasis and insulin sensitivity in *Seipin*^−/−^ mice and control mice at 6 months of age. *Seipin*^−/−^ mice showed impaired glucose tolerance test (GTT, Figure 1E). Insulin tolerance test (ITT) was performed and the result indicatedthat *Seipin*^−/−^ mice showed impaired insulin sensitivity compared with control mice (Figure 1F). They had significantly elevated fed plasma insulin levels, also demonstrated insulin resistance.

### Reduced PVAT Mass in *Seipin*^–/–^ Mice

**Figure 2:**
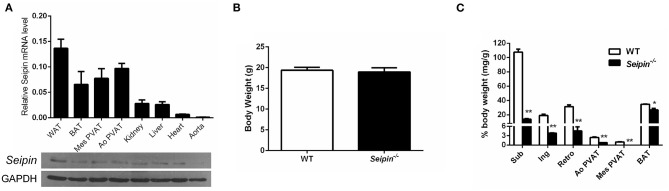
Reduced PVAT mass in *Seipin* deleted mice. **(A)** Detection of *Seipin* mRNA and protein expression in various tissues of WT mice. *N* = 6. **(B)** Body weight and **(C)** Mass of fat pad and PVAT in 6-month-old *Seipin*^−/−^ and WT mice. Brown adipose tissue (BAT), White adipose tissue (WAT), aortic perivascular adipose tissue (Ao PVAT), mesenteric PVAT (Mes PVAT). Data are expressed as mean ± SEM. *N* = 6. ^*^P < 0.05, ^**^P < 0.01 for *Seipin*^−/−^ mice vs. WT.

As revealed by real-time PCR and immunoblotting, *Seipin* is highly expressed in adipose tissue, including white adipose (WAT), brown adipose tissue (BAT), mesenteric PVAT (Mes PVAT) and aortic PVAT (Ao PVAT), but in negligible amounts in aorta (Figure 2A). Consistent with our previous findings in animals, there was no significant difference in body weight in 6-month-old *Seipin*^−/−^ mice and control WT mice (Figure 2B), and fat depots were dramatically reduced in the *Seipin*^−/−^ mice (Figure 2C). Compared with abundant PVAT surrounding aorta in WT mice, the PVAT weight of *Seipin*^−/−^ mice aortas was decreased to a significantly lower level, and there was rarely PVAT around mesenteric arteries (Figure 2C).

### Increased Inflammation, ER Stress and Fibrosis in PVAT of *Seipin*^–/–^ Mice

**Figure 3:**
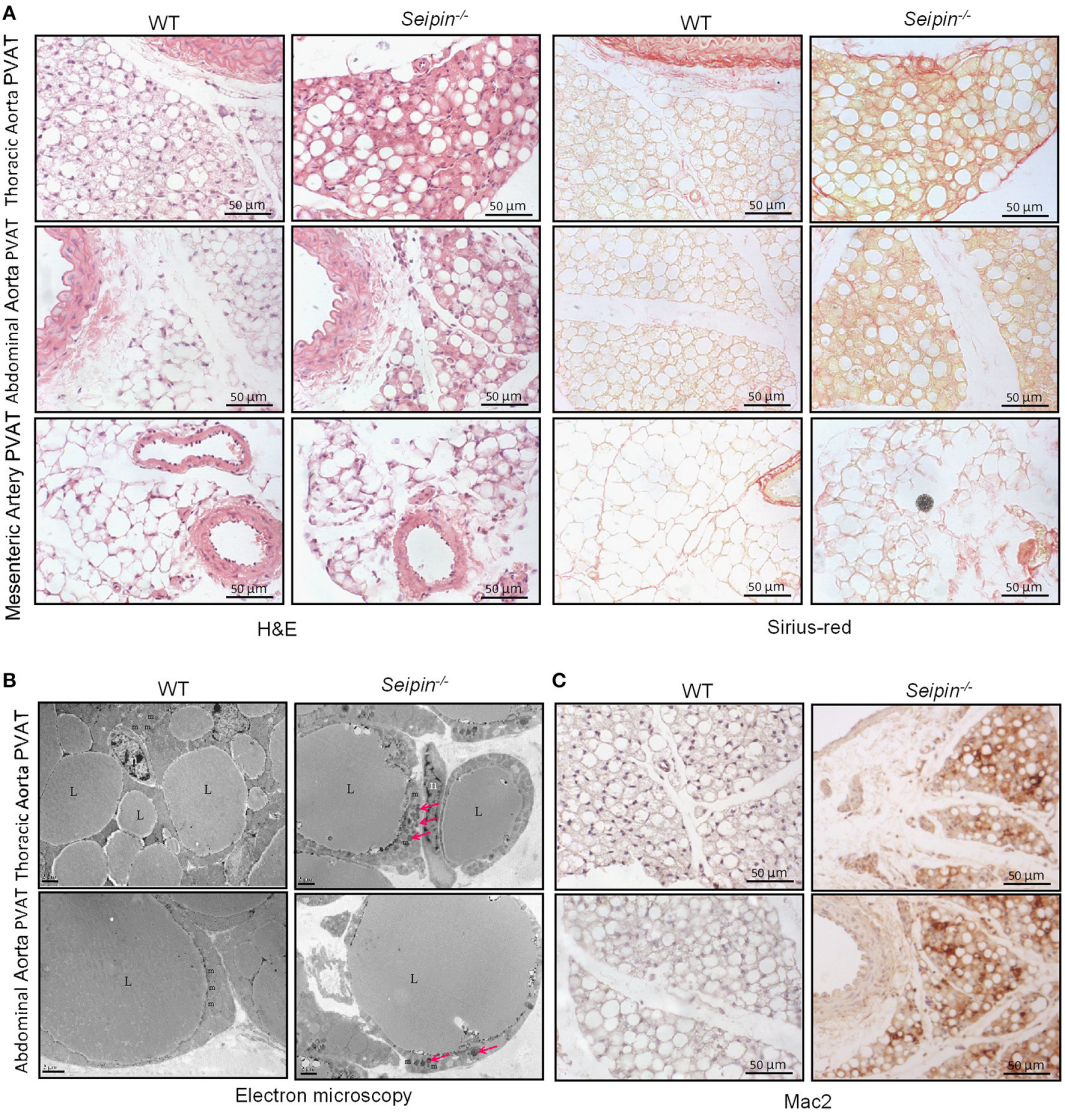
Histological observation of PVAT from WT and *Seipin*^−/−^ mice. **(A)** Representative H&E and sirius-red staining of PVAT from 6-month-old WT and *Seipin*^−/−^ mice. **(B)** Representative electron microscopy of PVAT from 6-month-old WT and *Seipin*^−/−^ mice. **(C)** Mac2 immunostaining of PVAT from 6-month-old WT and *Seipin*^−/−^ mice. m, mitochondria; n, adipocyte nucleus; L, lipid droplets. Pink arrow indicated deformed mitochondria.

**Figure 4:**
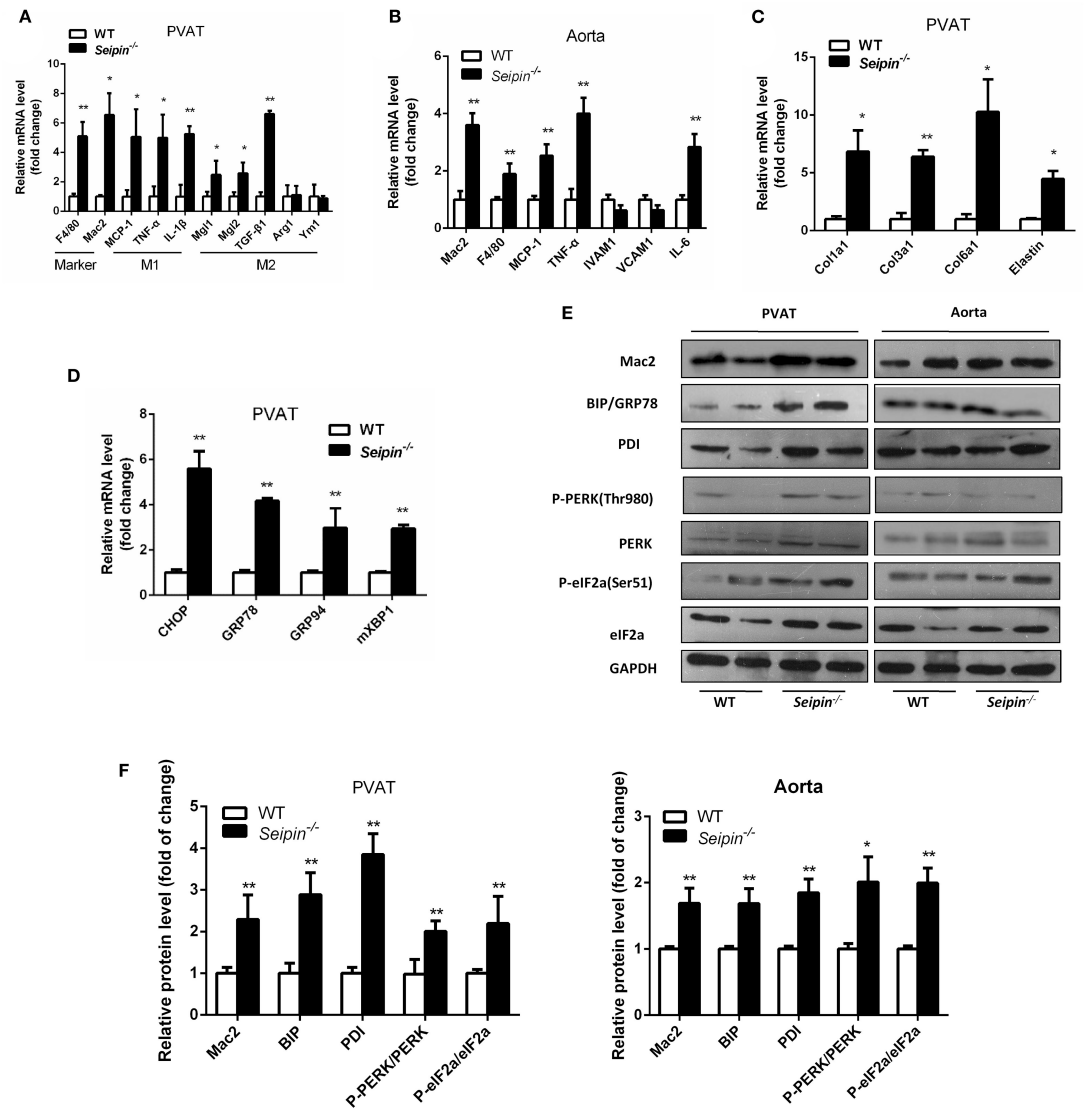
Inflammation, fibrosis and ER stress gene expression in PVAT and aorta from WT and *Seipin*^−/−^ mice. Relative mRNA levels of macrophages and inflammatory cytokines **(A,B)**, fibrosis **(C)**, and ER stress **(D)** in PVAT and aorta from 6-month-old WT and *Seipin*^−/−^ mice. *N* = 6. **(E,F)** Western blot images and densitometric quantitation for the indicated proteins and phosphoproteins related to inflammation and ER stress in PVAT from WT and *Seipin*^−/−^ mice. Data are expressed as mean ± SEM. *N* = 6, ^*^P < 0.05, ^**^P < 0.01 for *Seipin*^−/−^ mice vs. WT.

In haematoxylin and eosin stained sections, significant differences were observed in PVAT between WT and *Seipin*^−/−^ mice. In WT mice, thoracic PVAT had the appearance of BAT feature with small and multilocular lipid droplets, and mesenteric PVAT showed features of WAT with large single lipid droplets, whereas abdominal PVAT showed features of both WAT and BAT. Thoracic PVAT of *Seipin*^−/−^ mice were comprised of large, unilocular vacuoles similar to white adipocytes (Figure 3A). Interestingly, both thoracic and abdominal PVAT from *Seipin*^−/−^ mice were infiltrated with massive mononuclear cells, and displayed increased eosinophilic material (Figure 3A). As demonstrated by electron microscopy, we find that thoracic and abdominal PVAT of WT mice contains multilocular lipid droplets and mitochondria swollen with low dense matrices, whereas thoracic and abdominal PVAT of *Seipin*^−/−^ mice were composed of large lipid droplets and mitochondria that were spherical, large and packed with laminar cristae (Figure 3B bottom right, pink arrow). Mac-2 stained macrophages were abundant in thoracic and abdominal PVAT from *Seipin*^−/−^ mice (Figure 3C). Consistent with the histological observations, marker of macrophages, Mac2 and F4/80 gene expression was significantly increased in PVAT of *Seipin*^−/−^ mice (Figure 4A). In PVAT of *Seipin*^−/−^ mice, proinflammatory M1 and prorepair M2 macrophages-associated genes were both significantly elevated (Figure 4A). These findings demonstrated that PVAT of *Seipin*^−/−^ mice displayed increased inflammation. In aorta tissue, Mac2, F4/80, monocyte chemotactic protein 1 (MCP-1), TNF-α and IL-6 expression also elevated in *Seipin*^−/−^ mice (Figure 4B). Immunoblotting showed increased expression of Mac2 in PVAT and aorta of *Seipin*^−/−^ mice (Figures 4E,F). Additionally, analysis of collagen content, following Sirius-red staining demonstrated fibrosis apparently increased in PVAT of *Seipin*^−/−^ mice (Figure 3B). Corresponding to morphological results, fibrosis related genes were upregulated in PVAT of *Seipin*^−/−^ mice (Figure 4C). These findings reflected chronic inflammation and fibrosis were increased in PVAT of *Seipin*^−/−^ mice. Quantitative PCR revealed a high expression of the ER stress related gene CHOP, GRP78, GRP94, and mXBP1 (Figure 4D) in PVAT of *Seipin*^−/−^ mice. Consistent with the RNA data, immunoblotting results showed ER stress was activated in PVAT and aorta of *Seipin*^−/−^ mice (Figures 4E,F).

### Vascular Lesions in *Seipin*^–/–^ Mice

**Figure 5:**
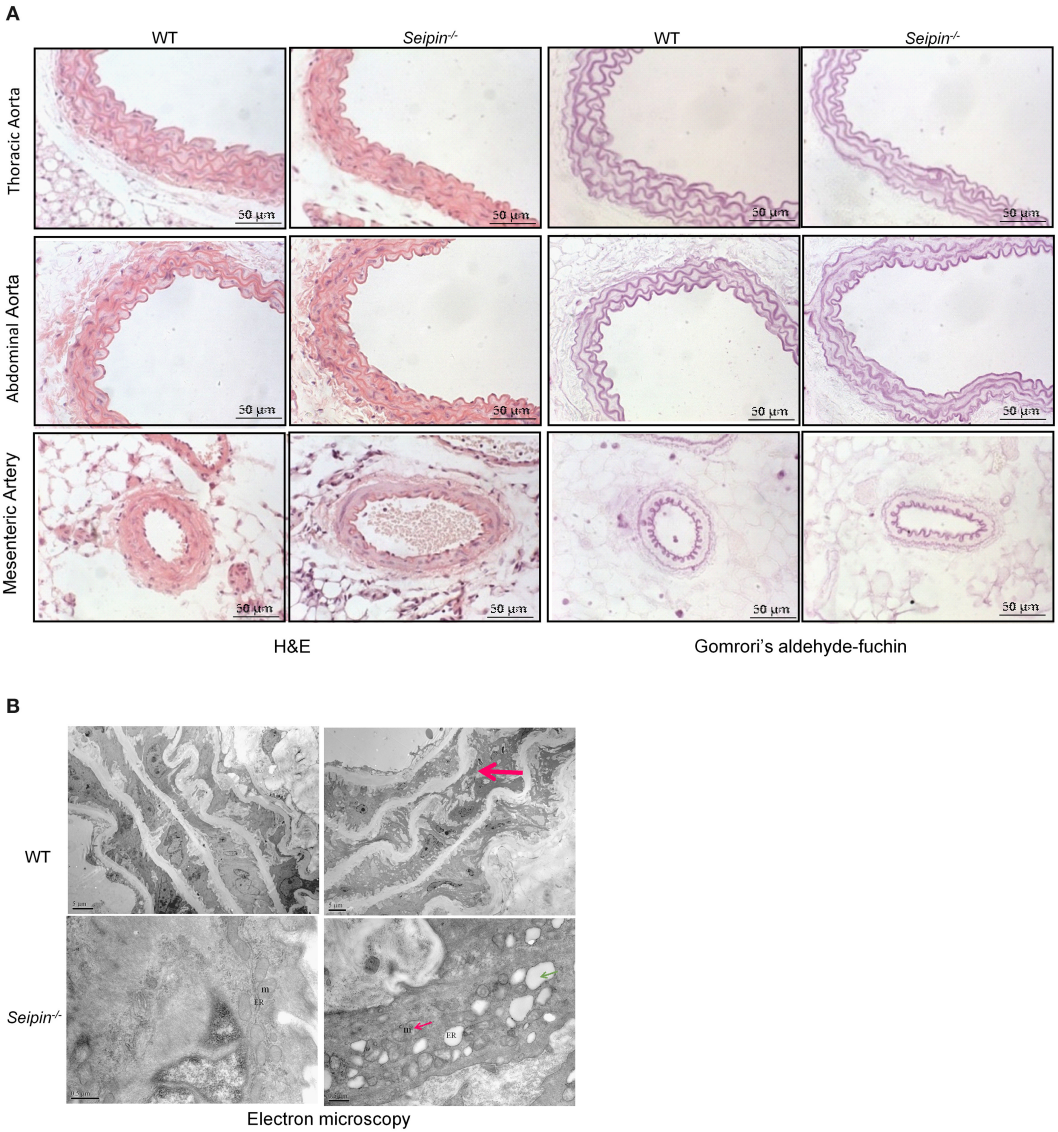
Vascular lesion in aorta of *Seipin*^−/−^ mice. **(A)** Representative H&E and Gomrori's aldehyde-fuchin staining in thoracic aorta, abdominal aorta and mesenteric artery in WT and *Seipin*^−/−^ mice. **(B)** Electron microscopy of thoracic aorta from 6-month-old *Seipin*^−/−^ and WT mice. Red arrow indicated derangement of elastic fibers in aorta. Thin red arrow showed deformed mitochondria. Green arrow indicated dilated ER. m, mitochondria; ER, endoplasmic reticulum.

Representative Gomrori's aldehyde-fuchsin staining showed derangement of elastic fibers in aorta of 6-months-old *Seipin*^−/−^ mice (Figure 5A top right, red arrow). The mitochondria with tubular cristae and rough surfaced endoplasmic reticulum were observed in normal mice aortas. Representative transmission electron microscope results showed disintegrating elastic laminae, swellen mitochondria and dilated rough endoplasmic reticulum in aorta of 6-month-old *Seipin*^−/−^ mice (Figure 5B bottom right, pink arrow and green arrow, respectively).

### Reduced Constriction and Impaired Endothelium-Dependent Relaxation in *Seipin*^–/–^ Mice

**Figure 6:**
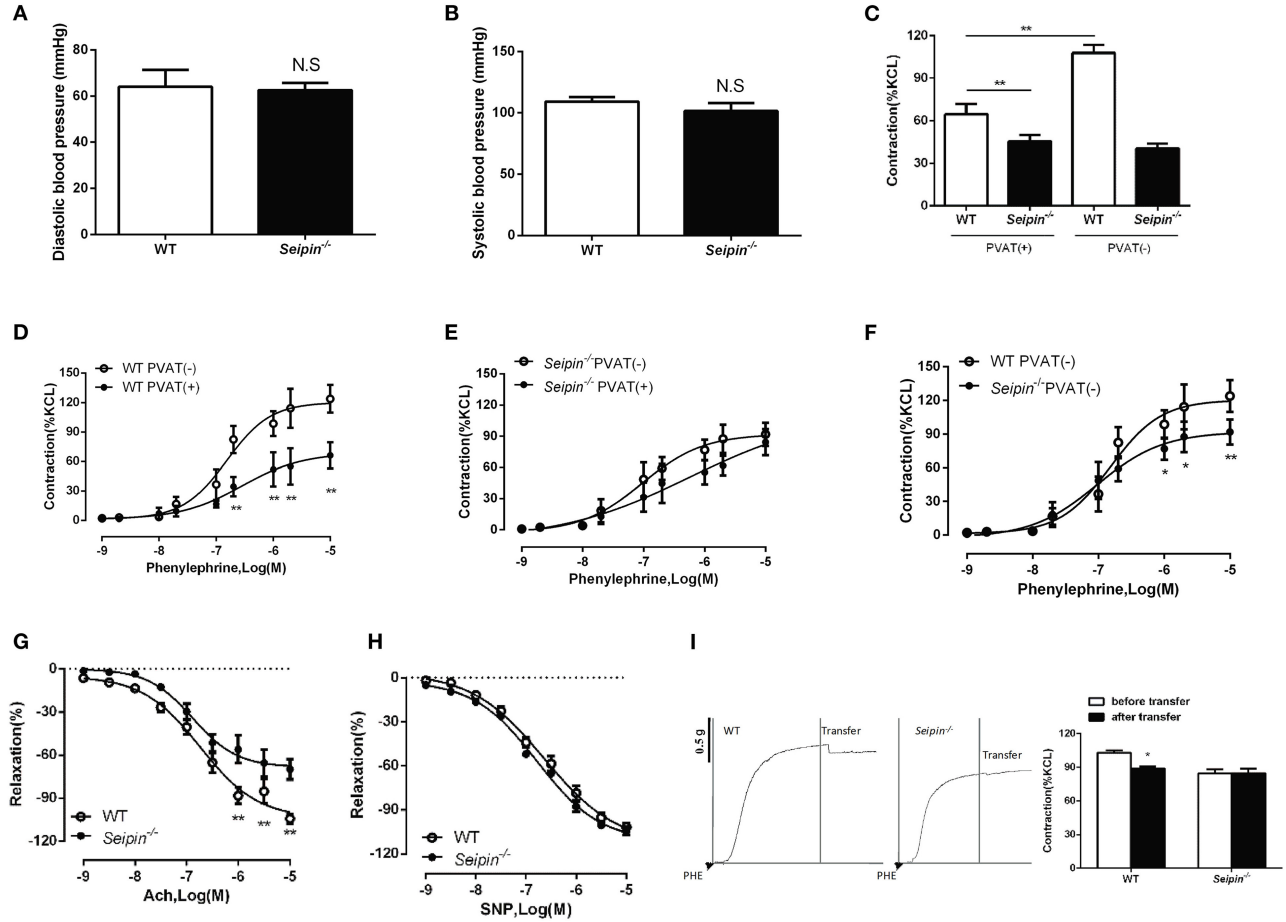
Impaired vasoactivity in *Seipin*^−/−^ mice. Systolic **(A)** and diastolic **(B)** of WT and *Seipin*^−/−^ mice. *N* = 10. **(C)** Phenylephrine induced contraction of aorta rings with (+) or without (-) PVAT prepared from 6-month-old WT and *Seipin*^−/−^ mice. **(D, E, F)** Phenylephrine dose-response curves of mesenteric artery rings with (+) or without (-) PVAT prepared from 6-month-old WT and *Seipin*^−/−^ mice. **(G, H)** Endothelial-dependent relaxation with acetylcholine (Ach) and smooth muscle relaxation with sodium nitroprusside (SNP) in aortic rings prepared from 6-month-old WT and *Seipin*^−/−^ mice. *N* = 10. **(I)** Representative curves of transferring 5 mL incubation of aorta PVAT of WT and *Seipin*^−/−^ mice to aorta without PVAT after preconstruction with phenylephrine (PHE, 10 uM). ^*^P < 0.05, ^**^P < 0.01 for *Seipin*^−/−^ mice vs. WT.

Lipodystrophy is associated with hypertension in humans (28, 29), we therefore sought to measure blood pressure in *Seipin*^−/−^ mice. There was no distinct difference in both systolic and diastolic blood pressure between WT and *Seipin*^−/−^ mice (Figures 6A,B). Additionally, the heart rates were similar between WT and *Seipin*^−/−^ mice (data not shown). Thenthe responses of aortic rings to graded levels of phenylephrine (PE) and acetylcholine (Ach) and sodium nitroprusside (SNP) were measured. Aortic and mesenteric artery rings prepared with or without *Seipin*^−/−^ PVAT showed a reduced constriction in response to PE (Figures 6C,F). In addition, compared with WT mice, *Seipin*^−/−^ mice showed impaired endothelium-dependent relaxation responses to Ach, but direct smooth-muscle relaxation in response to SNP was not altered (Figures 6G,H).

### *Seipin*^–/–^ Mice PVAT Lost the Anticontractile Effect

In WT mice, thoracic aorta and mesenteric artery rings contracted significantly less when PVAT was left, so WT PVAT exerted a normal anticontractile effect on the arteries (Figures 6C,D). However, in *Seipin*^−/−^ mice, the contractility of thoracic aorta and mesenteric artery rings with or without PVAT was similar, which suggest *Seipin*^−/−^ PVAT lost the anticontractile effect (Figures 6C,E). ADRFs play a critical role in PVAT anticontractile effect, and transferring culture medium experiments demonstrated that *Seipin*^−/−^ PVAT showed reduced ADRFs release (Figure 6I).

## Discussion

The aim of this study was to investigate the role of *Seipin* in PVAT function and vascular homeostasis. In present study, we found that *Seipin* deficiency induced increasing inflammatory factor secretion, macrophage infiltration, and ER stress activation inaddition to PVAT mass reduction; decreasing adiponectin, leptin and ADRF secretion resulting in PVAT anticontractile effect reduction. The alterations in PVAT morphology and function induced vessel ER stress and chronic inflammation and then *Seipin*^−/−^ mice displayed impaired contractility in response to phenylephrine and relaxation to acetylcholine.

*Seipin*^−/−^ mice represent probably the best model for lipodystrophy, and present a severe loss of adipose tissue, fatty liver and insulin resistance. However, little is known about whether lipodystrophy in *Seipin*^−/−^ mice is associated with hypertension, In this study, we demonstrated that the systolic and diastolic blood pressure was comparable between lipodystrophic *Seipin*^−/−^ mice and their control mice, which was consistent with previous research (30). Although lipodystrophy is associated with hypertension in humans (31, 32), previous studies have demonstrated that hypertension is separable from lipodystrophy in mice. A lipodystrophy model, A-ZIP/F mice, which lost almostwhite adipose tissue and showed dramatically reduced brown adipose tissue, were hypertensive (33, 34). In contrast, another lipodystrophy model MORE-PGKO, which is a generalized PPARγ knockout mouse, were hypotensive (35). Although, these findings suggest a controversial relationship between hypertension and lipodystrophy, lipodystrophic mice demonstrated PVAT dysfunction and impaired vasoactivity (27, 35). In mouse model, PPARγ deficiency reduced increased vascular relaxation and impairedcontraction. A-ZIP/F mice showed enhanced response of the blood vessels to agonists (34). Perilipin1 deficient mice demonstrated impaired anticontractile effect of PVAT and impaired endothelium-dependent vasodilatation (27).

Almost all systemic arteries were surrounded by a substantial amount of PVAT. Different from WAT and BAT, it is considered as active tissue by secreting numerous vasoactive yet-unidentified ADRFs. In addition to serving as mechanical support, PVATphysiologically antagonizes vasocontractile response to various vasoconstrictors (36, 37). Adiponectin and leptin were identified as abundant adipokines with anticontractile activity in recent studies (18, 38, 39). In *Seipin*^−/−^ mice, aortic and mesenteric PVAT showed obvious reduction in mass and adiponectin and leptin release, which provided histological and functional basis for impaired anticontractile effect. *Seipin* itself or its deficiency probably not have a direct effect *in situ* on vessels, in consideration of the low expression of *Seipin* in vascular endothelial and smooth muscle cells. *Seipin* is an abundant adipocyte protein, thus we speculated that impaired vascular homeostasis in *Seipin*^−/−^ mice was probablya consequence of perivascular adipose tissue dysfunction.

*Seipin* is an ER membrane protein and abundant in adipose tissue, testes and the brain. Recent studies have shown that *Seipin* depletion induces ER stress activation through influencing the intracellular calcium homeostasis in Drosophila fat cells and hepatocytes (5, 40). Another study reported that increased ER stress induced heart failure in *Seipin* deficient mice (9). In our study, *Seipin*^−/−^ mice also demonstrated increased ER stress in PVAT. ER stress is manifested in adipose dysfunction and which has been demonstrated to be involved in many pathological processes, including inflammation, oxidative stress and cell death. Our study demonstratedthat activated ER stress led to inflammation in *Seipin*^−/−^ PVAT.

In the present study, *Seipin*^−/−^ PVAT showed increased macrophage infiltration. Mac2-stained macrophages were prominent in PVAT of *Seipin*^−/−^ mice. Numerous macrophages especially M1 categories were present in PVAT from *Seipin*^−/−^ mice. M1 macrophages are characterized with pro-inflammatory properties. And expression of pro-inflammatory M1 macrophage-associated genes (TNF-α and MCP-1) was significantly upregulated in PVAT of *Seipin*^−/−^ mice compared to WT mice. Similarly, obvious infiltration of mac2 and F4/80-positive macrophages and increased inflammatory cytokines (TNF-α, MCP-1 and IL-6) were demonstrated in the aorta of *Seipin*^−/−^ mice.

Anticontractile effect of PVAT is involved many complicated processes including macrophage function. It's known that macrophage activation plays a critical role in the adipose tissue microenvironment, and is responsible for the loss of anticontractile function in inflamed PVAT (41, 42). PVAT-derived inflammatory cytokines such as TNF-α, MCP-1 and IL-1β impair PVAT anticontractile properties and vascular function in hypertension (43, 44). In *Seipin*^−/−^ mice, aortas and mesenteric arteries with or without PVAT had similar vasoconstriction responses, which suggested *Seipin*^−/−^ PVAT lost anticontractile function. *Seipin*^−/−^ PVAT displayed extensive macrophage infiltration, inflammatory adipokines expression and activated ER stress. And *Seipin*^−/−^ aorta also demonstrated increased MCP-1 and IL-6 expression. Collectively, these multiple culprits could contribute to the vascular lesions in *Seipin*^−/−^ mice. Vascular lesions including derangement and fragmentation of elastic fibers might contributed to reduced constriction in response to PE.

In conclusion, *Seipin* deficiency induced PVAT mass reduction and ADRFs secretion, along with abnormal morphology, macrophages infiltration and activated ER stress, resulting in impairedanticontractile effect of PVAT. In *Seipin*^−/−^ mice, PVAT dysfunction induced vessel chronic inflammation, ER stress and vascular lesions. As a result, vessels in *Seipin*^−/−^ mice had impaired contractility in response to phenylephrine and relaxation to acetylcholine.

## Data Availability Statement

The original contributions presented in the study are included in the article/supplementary material, further inquiries can be directed to the corresponding author/s.

## Ethics Statement

The animal study was reviewed and approved by Animal Care Committee of Zhengzhou University.

## Author Contributions

MW and JX performed the experiments and data analysis. ML, MG, YL, XL, and LH participated in the collection of samples and data. XZ and JL provided technical advice. GL and JD designed the study and wrote the grant application. All authors were involved in writing the manuscript. The manuscript is an original work and the final version has been read and approved by all authors.

## Conflict of Interest

The authors declare that the research was conducted in the absence of any commercial or financial relationships that could be construed as a potential conflict of interest.

## Publisher's Note

All claims expressed in this article are solely those of the authors and do not necessarily represent those of their affiliated organizations, or those of the publisher, the editors and the reviewers. Any product that may be evaluated in this article, or claim that may be made by its manufacturer, is not guaranteed or endorsed by the publisher.
